# Congenital Megaprepuce: Literature Review and Surgical Correction

**DOI:** 10.1155/2019/4584609

**Published:** 2019-05-14

**Authors:** Zachary Werner, Ali Hajiran, Osama Al-Omar

**Affiliations:** Suite 6300, Health Sciences Center, Morgantown, WV 26506, USA

## Abstract

Congenital megaprepuce (CMP) is a type of buried penis characterized by extensive redundancy and ballooning of the inner prepuce as a result of preputial stenosis and phimosis. The malformation typically presents with difficulty voiding, often requiring manual expression of stagnant urine. Multiple techniques have been reported for the treatment of CMP with varying levels of positive outcomes. The authors provide a review of published literature, in addition to describing the procedure and results of our surgical technique in three children aged eleven months, two years, and twelve years. The literature review was conducted using PubMed with keywords “congenital megaprepuce,” “megaprepuce,” “buried penis,” “CMP,” and “correction.” Results were then differentiated based on presence or absence of true congenital megaprepuce and the surgical correction thereof. Regarding our cases, all patients completed the procedure with excellent cosmesis and without complication. Our technique is shown to provide consistent, excellent esthetic outcome across a wide range of ages and may be replicated by others.

## 1. Introduction

Congenital megaprepuce is a subtype of buried penis. Maizels et al. defined buried penis as a penis of normal size hidden by preputial skin [1]. We conducted a literature review of techniques and cases using this definition as a guideline. It differs from various other forms of buried penis including webbed penis, buried penis secondary to circumcision, secondary to large suprapubic fat pad, and secondary to large hernia or hydrocele. Although Summerton et al. theorized that phimosis played little role in the abnormality, multiple modern authors refute this [2–5]. Congenital megaprepuce typically presents with a stenotic preputial ring with inner preputial ballooning as a result of the hydrostatic pressure of accrued urine [4, 6, 7]. Multiple sources have suggested that the etiology of this condition is an abnormal development of the migrational planes of the penis resulting in decreased ventral skin, dysplastic dartos fascia, and improper anchoring of the skin at the base of the penis [7–9].

The condition can present as early as birth but commonly is diagnosed later in infancy, most often due to parental concern regarding the need for manual urine expression and abnormal appearance [3, 6, 7, 10, 11]. While infants often present with benign micturition difficulties without lasting effects, adolescents with CMP may suffer psychological damage both from appearance and difficulty with penetration [4, 10]. Most authors assert that CMP should be treated surgically, often soon after diagnosis [3, 6, 10, 11]. Circumcision is contraindicated in these children as the preputial skin is required for repair.

Various methods have been used for the correction of CMP including the ventral v-plasty, Cuckow's technique, preputial unfurling, and a multitude of other methods; however, there is no “gold-standard” or consensus of the most efficacious technique [2, 3, 5–8, 10, 11]. Here, we describe our surgical strategy, which produced excellent cosmesis and repair.

## 2. Case Descriptions

Three cases of CMP, aged eleven months, two years, and twelve years, were surgically treated in 2017-2018 at our institution via excision of excess preputial skin, penile reconstruction, and scrotoplasty.

The eleven-month-old had a history of preterm birth at 24 weeks. He underwent unsuccessful circumcision at an outside facility and was sent to us for difficulty with micturition with ballooning and manual expression, in addition to requirement of circumcision revision. Upon examination, he was noted to have a phimotic ring and right-sided cryptorchidism.

**Figure 1:**
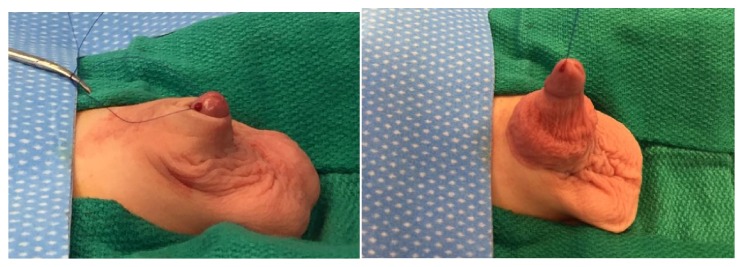
(a) shows the buried penis in normal position. (b) further details the extent of the excess inner prepuce.

The two-year-old presented to the Urology clinic with difficulty of micturition and ballooning, sometimes requiring parental manual expression of foul urine. Upon examination, he was noted to have severe phimosis and buried penis with a large amount of trapped urine (Figures 1(a) and 1(b)).

The twelve-year-old presented to the Urology clinic primarily for circumcision. He also described the need for postvoiding manual expression of urine.

### 2.1. Surgical Technique

**Figure 2:**
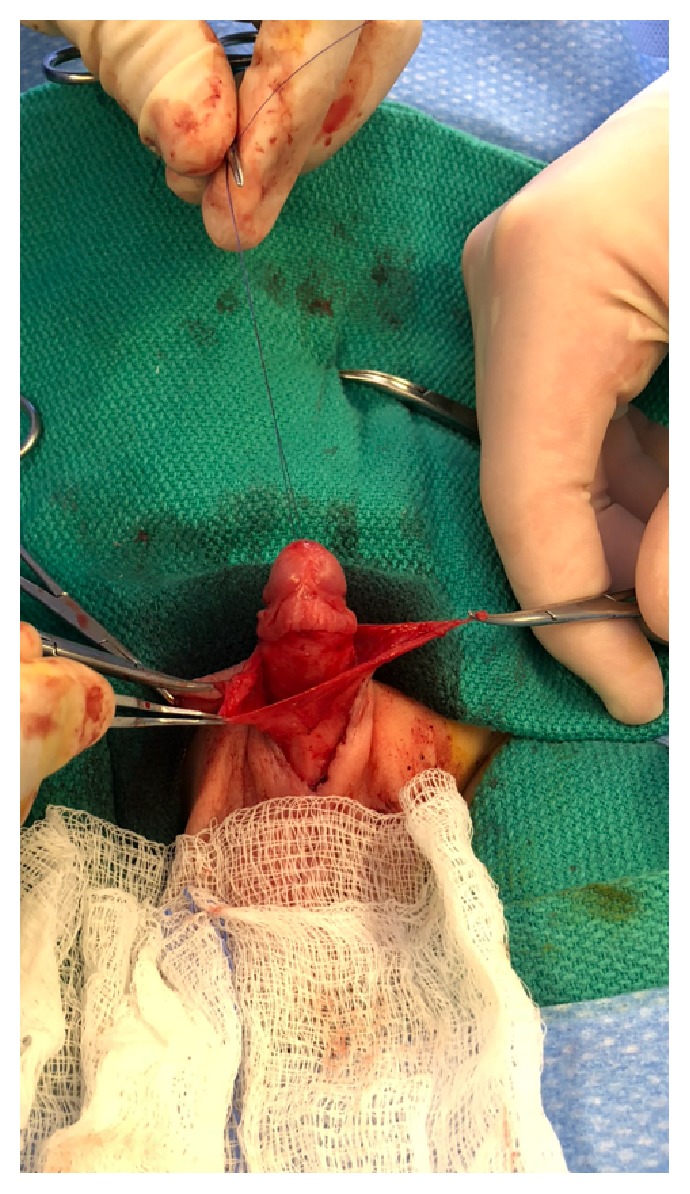
Isolated dysplastic dartos muscle prior to excision.

**Figure 3:**
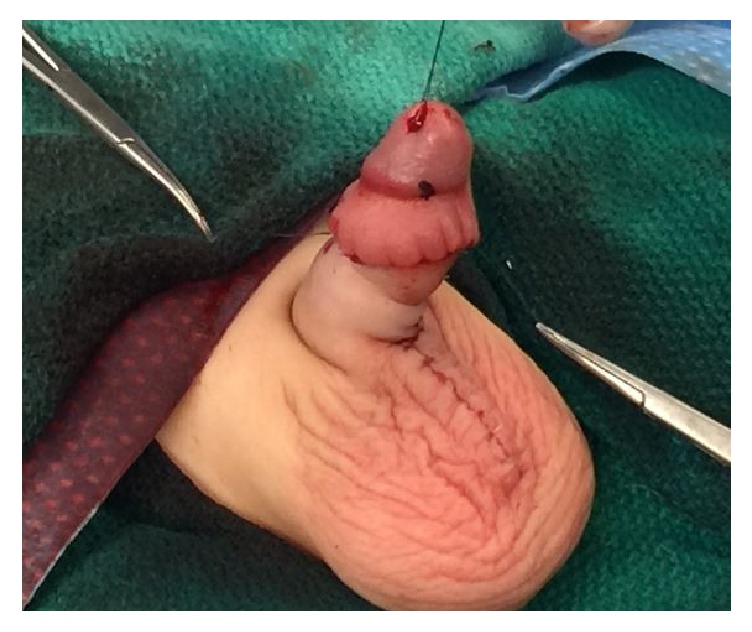
Good recreation of the penoscrotal and penopubic angle is shown, in addition to scrotal reapproximation at the midline.

**Figure 4:**
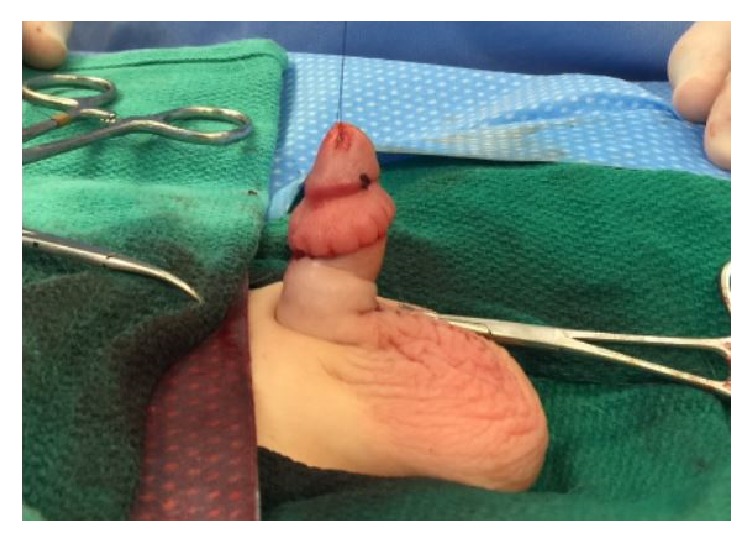
Redemonstration of the penopubic and penoscrotal angles in the two-year-old patient.

All surgeries were performed under general anesthesia. Using a hemostat, the glans of the penis was exposed by ventrally or dorsally opening the phimotic ring. A 5.0 PDS stitch was placed in the glans for intraoperative control of the penile shaft, as shown in Figure 1(b). The key steps in the surgery are as follows:

1. Radical degloving of the penis: a circumferential incision 10 mm from the coronal sulcus was made. The penile shaft skin then incised ventrally and longitudinally down to the penoscrotal junction in order to radically deglove the penis to Buck's fascia until reaching the penile base deep in the scrotum.
2. Radical excision of the dysplastic and thick dartos muscle down to the base of the penis (Figure 2).
3. Excision of the redundant inner prepuce and preparing the penile shaft Byer skin flaps, which were rotated ventrally in order to have sufficient penile shaft skin coverage.
4. Recreation of the penopubic and penoscrotal angles by using two PDS anchoring stitches dorsally and ventrally, retrospectively, between the dermis and Buck's fascia at the dorsal and ventral base of the penis. The scrotum was reapproximated in two layers at the midline using 4.0 Monocryl sutures and the ventral penile skin was similarly reapproximated at the ventral midline and circumferentially as shown in Figures 3 and 4.

All patients completed the procedure with excellent cosmesis. No immediate postoperative complications such as infection, bleeding, or urinary retention were observed.

**Figure 5:**
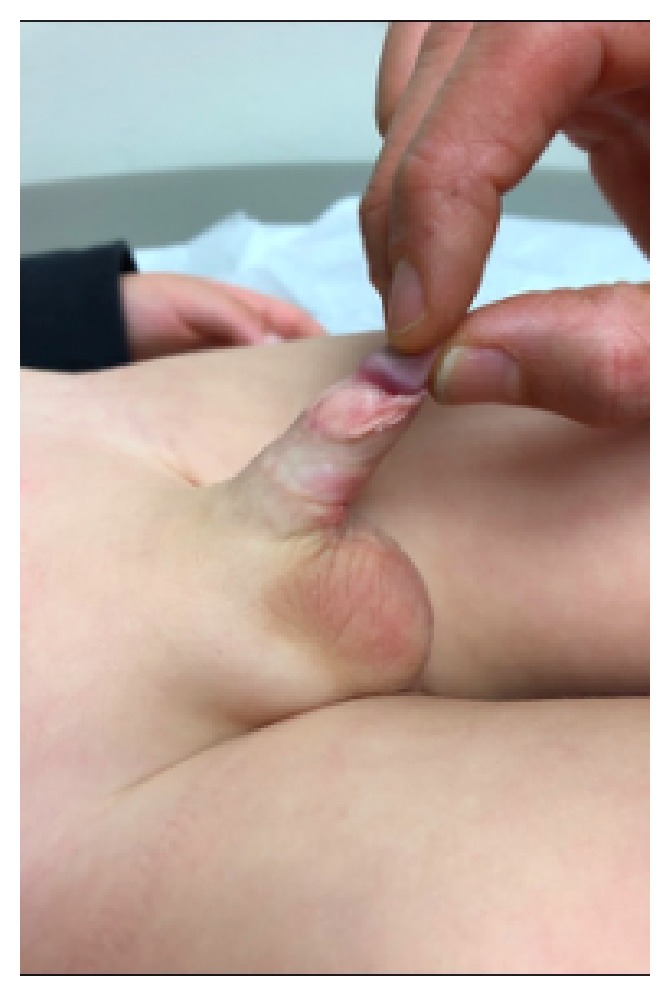
Demonstration of excellent cosmesis at eleven-month follow-up.

The two-year-old boy showed excellent cosmesis and parental satisfaction at eleven-month follow-up visit (Figure 5).

Unfortunately, the twelve-year-old and eleven-month-old failed to appear for follow-up and cannot be reached for short-term results.

## 3. Discussion

Discussion regarding the condition of congenital megaprepuce can be especially confusing given the ill-defined nomenclature surrounding the disease. The first case of congenital megaprepuce was described in 1994 [12]. Various terms such as buried penis, trapped penis, webbed, concealed, or entrapped were all seemingly used interchangeably in the past. We use the definitions defined by Maizels et al. which defines buried penis as a subtype of concealed penis [1]. Further, we consider congenital megaprepuce to be describing a childhood disease characterized by phimosis and ballooning of the inner prepuce [5, 6]. Congenital megaprepuce is a condition best treated by surgical correction, often at the time of diagnosis. There is an abundance of literature and techniques for describing and treating general buried penis [13–25]. However, upon a review of the literature, we found there is significantly less literature regarding specific surgical correction of CMP.  Table 1 lists the various articles found upon review that are specific to CMP, many of which contain techniques for general buried penis that have been repurposed for the treatment of CMP [2, 3, 5–8, 11, 26–33]. The majority of the papers describe incision of the stenotic ring, unfurling of the prepuce with subsequent inner preputial resection, followed by a variety of resurfacing techniques [2, 3, 5–8, 11, 26–33].

Our method of correction provided similar levels of cosmetic repair in patients of very different ages and is thus applicable across a wide spectrum of patients. The technique reaffirms some of the most important aspects of repair including removal of the stenotic ring, excision of redundant inner preputial skin, and anchoring of the skin to Buck's fascia to recreate the penopubic and penoscrotal angles. However, we believe that the most important step in correcting CMP is excising the dysplastic dartos muscle which helps achieve excellent penile skin reconstruction, penoscrotal angle, penopubic angle, and penile shaft skin approximation, based on our limited case series. Leaving the dartos muscle results in downward retraction of the penile skin with thick and poor cosmesis of the penile skin reapproximation. This critical step is described in the majority of papers reviewed.

Although the ventral v-plasty technique described by Alexander et al. has shown good results, we believe a dorsal skin flap provides excellent cosmetic repair [7, 11]. This sentiment was echoed by other authors who have similarly used dorsal skin to achieve a normal circumcised appearance [6, 11]. Additionally, we are proponents of penile coverage with the outer preputial skin with excision of the redundant inner prepuce, as replacement of the inner prepuce has been shown to cause postoperative edema and poor esthetics [2, 6, 7, 26]. It should be noted though that of the 134 patients treated by Chin et al. most of their patients' postoperative edema resolved by one month, although one patient did require a second surgery due to persistent swelling [27]. We did not induce artificial erection as penile degloving in our cases did not indicate any concern regarding penile chordee, although Liu et al. have made it part of their standard procedure [26].

One of the most important aspects of CMP is early recognition and proper age of surgical correction. Murakami et al. reported a common misbelief among parents that the patients would eventually grow out of the appearance of their penis [11]. Liu et al. recommended waiting until completion of toilet training to initiate surgical treatment [26]. Multiple sources dispute this recommendation, citing significant psychological damage with delayed surgical correction as an unneeded side effect [3, 6, 9, 10]. De Jesus et al. recommend surgery for buried penis between ages one and three to prevent psychological damage [10]. Despite CMP presenting in infancy, the average age at repair for our review was 24.5 months, although one can assume that age at initial presentation plays a large role in age at repair. We believe surgical correction is best conducted at time of initial diagnosis.

Given the rarity of this disease, it is difficult to have a sample size large enough for strong recommendations on technique. In addition to having a larger sample size to draw conclusions from, longer follow-up is needed to confirm parental and patient satisfaction, lack of recurrent buried penis, and general results of cosmesis and repair. In our humble series, the lack of follow-up with the twelve-year-old is especially disappointing, as he is one of the oldest presenting patients we found during our review of the literature.

## Figures and Tables

**Table 1:** Results of congenital megaprepuce literature review.

First author	Title	Cases	Minimum age	Maximum age	Average age
Ruiz	Simplified surgical approach to Congenital Megaprepuce. Fixing, Unfurling, and Tailoring Revisited	26	2 months	19 months	6 months

Alexander	The Ventral V-plasty: a simple procedure for the reconstruction of a congenital megaprepuce	10	8 months	3 years	20 months

Lin	An Arc Incision Surgical Approach in Congenital Megaprepuce	32	10 months	3 years	Unknown

Summerton	Congenital megaprepuce: an emerging condition. How to recognize and treat it	20	6 months	3.6 years	16 months

Rod	Congenital megaprepuce: a 12-year experience (52 cases) of this specific form of buried penis	52	4 months	2.3 years	13 months

Liu	Congenital completely buried penis in boys: anatomical basis and surgical technique	22	2.5 years	5.8 years	4.2 years

Chin	Modified prepuce unfurling for buried penis: a report of 12 years of experience	134	2 months	33 years	5.5 years

Shenoy	Surgical correction of congenital megaprepuce	3	3 months	6 months	5 months

Buluggiu	Congenital Megaprepuce: surgical approach	5	6 months	7 years	2.5 years

Murakami	A single surgeon's experience of 65 cases of penoplasty for congenital megaprepuce, with special reference to mid- to long-term follow-up	65	4.8 months	13.9 years	5.9 years

Leao	Congenital megaprepuce: a new alternative technique for surgical correction	5	2 years	5 years	Unknown

Delgado	Congenital megaprepuce: diagnosis and therapeutic management	4	Unknown	Unknown	Unknown

Callewaert	Double longitudinal megapreputium Incision Technique: the Dolomite	6	7 months	2.1 years	13.3 months

Podesta	Megaprepuce Reconstruction: A Single Center Experience	15	3 months	20 months	9 months

Serrano-Durba	Secondary congenital megaprepuce	1	Unknown	Unknown	3 months
